# Impact of Trace Minerals on Wound Healing of Footpad Dermatitis in Broilers

**DOI:** 10.1038/s41598-017-02026-2

**Published:** 2017-05-15

**Authors:** Juxing Chen, Guillermo Tellez, Jeffery Escobar, Mercedes Vazquez-Anon

**Affiliations:** 1 Novus International, Inc. 20 Research Park Drive, St, Charles, MO 63304 USA; 2 Department of Poultry Science, University of Arkansas, Fayetteville, AR 72701 USA; 3 Elanco Animal Health, 2500 Innovation Way, Greenfield, IN 46140 USA

## Abstract

Footpad dermatitis (FPD) is used in the poultry industry as an animal welfare criterion to determine stocking density. Trace minerals (TM) play a role in skin integrity and wound healing. This study evaluated the impact of TM on FPD and consisted of 3 treatments supplemented with 0 (NTM), low (LTM) and high (HTM) TM levels in the same basal diet. On d21, 71% birds in all treatments developed mild FPD and pens were top-dressed with dry litter to promote FPD healing. Compared to NTM, LTM reduced area under the curve (AUC) of FPD lesion scores during d21–42, HTM reduced the AUC of FPD lesion scores during d7–21 and d21–42. LTM improved growth performance on d14, HTM improved growth performance on d14 and d28. LTM and/or HTM increased gene expression of VEGF, TIMP3, TIMP4, MMP13, ITGA2, ITGA3 and CD40, which promoted collagen synthesis, deposition and organization; cell migration, matrix remodeling, and angiogenesis. LTM and/or HTM increased inflammation by upregulating TNFα and IL-1β during the early wound healing phase and reduced inflammation by downregulating IL-1β during the late wound healing phase. Our findings showed that TM not only improved growth performance but also reduced FPD development by promoting FPD wound healing.

## Introduction

Footpad dermatitis (FPD) is a skin inflammation that causes necrotic lesions on the plantar surface of footpads in poultry^1^. In some countries, chicken feet or paws are considered as gastronomic delicacy and at present constitute the third most important economic part of the chicken (following chicken breast and wings). Chicken paw profitability has been estimated at approximately $280 million a year^2^. In addition to causing increased condemnation loss, the incidence of footpad lesions is also an audit criterion in welfare valuations of poultry production^3, 4^. In addition, lesions in footpads can induce systemic infection in poultry, hence, FPD is considered as a food safety issue^4–6^. FPD is a multifactorial condition that is caused by dietary ingredients, nutritional deficiencies, wet litter, litter material types, genetic strain, sex, bird weight, stocking density, season, and management^4, 7, 8^. Wet litter has been reported to be the most central predisposing factor for FPD development in poultry^9–11^. Some researchers have reported that replacement of wet litter with dry litter improved the footpad lesions in about two weeks^8, 12^. Unfortunately, in the field, replacement of wet litter is not practical or economical, hence, alternative practices should be considered. Trace minerals (TM) such as Zn, Cu, and Mn are known to play a role in maintaining the structural integrity of different tissues including skin^6, 13–17^. Nutraceuticals such as probiotics, prebiotics, or enzymes that improve intestinal integrity and improve fecal consistency and litter quality can also reduce footpad lesions^18^. Recently, we identified a variety of biomarkers for footpad lesion development and wound healing which can be used to better understand the pathology and etiology of FPD, and find strategies to intervene or prevent the development of footpad lesions and promote the wound-healing process^19^. The objectives of this study were to evaluate the impact of TM on FPD development and wound healing.

## Methods

### Animals and Diets

A total of 324 hatchling Ross 308 male broiler chickens were randomly assigned to 3 treatments and housed in 2′ × 3′ floor pens in an environmentally controlled room. A 3 cm layer of chopped wheat straw was used as bedding material and applied uniformly in each pen across all treatments on the first day of the experiment. Temperature was maintained at 34 °C for the first 5 d and then gradually reduced according to normal management practices, until a temperature of 23 °C was achieved. Hours of light (L) and dark (D) were provided as follows: d 1 to 3, 23L:1D; d 4 to 12, 18L:6 D; d 13 to 30, 16L:8D. Birds were fed crumbled starter diets from d 0 to 14; switched to pelleted grower diets from d15 to 28, and then to pelleted finisher diets from d29 to 43 (Table 1). Birds had free access to feed and water at all times. Experimental diets were formulated to meet or exceed nutritional requirements of broiler chickens with the exception of TM^20^. All research procedures were reviewed and approved by the animal ethics committee composed of members from Novus International Inc (20 Research Park Drive, St. Charles, MO 63304) and a licensed veterinarian from Bridgeton Animal hospital (3148 McKelvey Road, Bridgeton, MO 63044). All studies performed by Novus International, Inc. are in accordance with the standards of the Guide for the Care and Use of Agricultural Animals in Research and Teaching^21^.

**Table 1:** Ingredient composition and nutrient content of the experimental diets.

Item	Starter (d 0–14)			Grower (d15–28)			Finisher (d29–43)		
	NTM	LTM	HTM	NTM	LTM	HTM	NTM	LTM	HTM
Wheat	47.30	47.14	47.18	45.17	45.01	45.05	50.77	50.61	50.65
Soybean meal, 47.5% CP	33.40	33.40	33.40	29.36	29.36	29.36	23.90	23.90	23.90
Barley	10.00	10.00	10.00	15.00	15.00	15.00	15.00	15.00	15.00
Soybean oil	4.45	4.45	4.45	6.30	6.30	6.30	6.30	6.30	6.30
L-lysine HCl	0.25	0.25	0.25	0.15	0.15	0.15	0.16	0.16	0.16
Methionine hydroxy analogue^1^	0.42	0.38	0.34	0.35	0.31	0.27	0.32	0.28	0.24
L-threonine	0.11	0.11	0.11	0.07	0.07	0.07	0.07	0.07	0.07
Dicalcium phosphate 18.5%	1.80	1.80	1.80	1.55	1.55	1.55	1.45	1.45	1.45
Limestone	1.25	1.25	1.25	1.05	1.05	1.05	1.05	1.05	1.05
Salt	0.50	0.50	0.50	0.50	0.50	0.50	0.50	0.50	0.50
Choline cloride-60%	0.27	0.27	0.27	0.25	0.25	0.25	0.23	0.23	0.23
Mineral premix	—	0.20^2^	0.20^3^	—	0.20^2^	0.20^3^	—	0.20^2^	0.20^3^
Antioxidant^4^	0.02	0.02	0.02	0.02	0.02	0.02	0.02	0.02	0.02
Fungicide^5^	0.05	0.05	0.05	0.05	0.05	0.05	0.05	0.05	0.05
Coccidiostat^6^	0.05	0.05	0.05	0.05	0.05	0.05	0.05	0.05	0.05
Bacitracin methylene disalicylate^7^	0.03	0.03	0.03	0.03	0.03	0.03	0.03	0.03	0.03
Vitamin premix^8^	0.10	0.10	0.10	0.10	0.10	0.10	0.10	0.10	0.10
Calculated analysis									
ME, kcal/kg	3034	3034	3031	3155	3155	3153	3201	3199	3199
Crude protein, %	22.8	22.7	22.7	21	21	21	19	19	19
SID^9^ Lysine, %	1.27	1.27	1.27	1.1	1.1	1.1	0.97	0.97	0.97
SID Methionine,%	0.64	0.64	0.64	0.56	0.56	0.56	0.5	0.5	0.5
SID Sulfure amino acids, %	0.94	0.94	0.94	0.84	0.84	0.84	0.76	0.76	0.76
SID Threonine, %	0.83	0.83	0.83	0.73	0.73	0.73	0.65	0.65	0.65
Ca, %	1.05	1.05	1.04	0.9	0.9	0.89	0.86	0.86	0.85
Available phosphorus, %	0.5	0.5	0.5	0.45	0.45	0.45	0.42	0.42	0.42

^1^Methionine hydroxy analogue (MHA^®^) (Novus International, Inc., St. Charles, MO).

^2^Mineral premix for LTM treatment supplied per kilogram of diet: FeSO_4_·H_2_O, 40 mg; Calcium Iodate, 1.25 mg; Sodium Selenite, 0.3 mg; Mintrex^®^Zn, 32 mg; Mintrex^®^Cu, 8 mg; Mintrex^®^Mn, 32 mg.

^3^Mineral premix for HTM treatment supplied per kilogram of diet: FeSO_4_·H_2_O, 40 mg; Calcium iodate, 1.25 mg; Sodium selenite, 0.3 mg; Mintrex^®^Zn, 64 mg; Mintrex^®^Cu, 16 mg; Mintrex^®^Mn, 64 mg.

^4^Santoquin^®^ (Novus International, Inc., St. Charles, MO).

^5^Mold guard^®^ (Kemin, Des Moines, Iowa).

^6^Coban^®^90 (Elanco Animal Health, Greenfield, IN).

^7^BMD^®^ (Zoetis, Inc., Florham Park, NJ).

^8^Vitamin premix supplied per kilogram of diet: retinol, 9.2 mg; cholecalciferol, 100 µg; dl-α-tocopherol, 90 mg; menadione, 6 mg; thiamine, 6.2 mg; riboflavin, 26.5 mg; pantothenic acid, 39.7 mg; niacin, 100 mg; pyridoxine, 11 mg; folic acid, 4 mg; biotin, 0.3 mg; cyanocobalamin, 0.1 mg.

^9^Standardized ileal digestibility.

### Experimental Design

This study evaluated the impact of TM on FPD development and wound healing, and consisted of 3 treatments with the same barley-wheat based diet and 3 supplemental TM levels: 0:0:0 ppm (NTM), 32:8:32 ppm (LTM), 64:16:64 ppm (HTM) of methionine hydroxy-analogue chelate of Zn:Cu:Mn (Mintrex^®^, Novus International, Inc.). Fe, I, Se (40, 1.25, 0.3 ppm) were added to LTM and HTM treatments but not to NTM treatment. Each treatment had 9 replicate floor pens with 12 birds per pen. The same amount of chopped wheat straw (3 cm depth) was used as bedding material (litter) and applied uniformly in each pen across all treatments at the first day of the experiment. Litter samples to measure litter moisture (per pen basis in all treatments) were collected from similar spot at least 1 foot away from feeder and waterer/nipple drinker. The sample size was from 2–5 inches across with a depth of 3–9 cm (from the top to the hard floor). Sampling was done on d6, 16, 20, 26 and 33. On d21, 71% chickens developed mild to moderate footpad lesions. To promote FPD wound healing, fresh and dry litter of 2 cm depth was added on top of existing litter in each pen across all treatments on d21, 28 and 35. At d14, 28, and 42 body weight (BW), cumulative body weight gain (cBWG), cumulative feed intake (cFI), and cumulative feed conversion (cFCR) were recorded in each pen to calculate cumulative performance index (cPI) = (cumulative livability*BW*100/Day of study)/cFCR.

### Footpad Lesion Scoring and Sample Collection

At 7, 14, 21, 28, 35 and 42 d-of-age, right and left footpads from all birds in each pen were examined by the same scorer throughout the study. Footpads were scored for ulcer and scabs after dipping the feet in a bucket of water and removing all adhering material from the footpad, and scored using 5-point scoring system. The scoring system was as follows: Score 1 = no lesions, dermal ridges intact within central plantar footpad surface, with or without discoloration; Score 2 = mild lesions, dermal ridges not intact with a small scab (≤2 mm in diameter) on the central plantar footpad surface; Score 3 = moderate lesions with a scab of 2–7 mm in diameter on the central plantar footpad surface; Score 4 = severe lesions with a large scab of ≥7 mm in diameter in the central plantar footpad surface but no scabs on the toes; Score 5 = most severe lesions with a large scab of ≥7 mm in diameter in central plantar footpad surface and small scabs on the toes^8^ (Supplemental Figure 1). At 15, 22, 29, 36 and 43 d-of-age, one representative chicken from each pen with lesions closest to the pen average lesion score was chosen to collect footpad skin samples for total collagen protein and gene expression analyses.

### Total Collagen Analysis

One piece of the left footpad of each bird per pen was collected and flash frozen on dry ice for collagen analysis. Footpad skin hydroxyproline (Hyp) content was measured to estimate collagen content^22^ with some modifications. For the assay, 100 mg of tissue was homogenized in 6 ml of 6 N HCl in a glass tube using a Cole Parmer Lab Gen 700 (Cole-Parmer North America, Vernon Hills, IL) for about 30 sec, and hydrolyzed at 110 °C for 24 h after adding an additional 6 ml of HCl. One ml of hydrolyzed sample was neutralized with 2 ml of 3 N NaOH and diluted 2× with distilled H_2_O. Serial dilutions of 0.1 mg/ml trans-4-hydroxy-L-proline were used as standard to generate standard curve. A 0.25-ml aliquot of 0.01 M CuSO_4_, 0.25 ml 2.5 N of NaOH, and 0.25 ml of 6% H_2_O_2_ were added in succession into glass tubes with 0.5 ml of diluted samples or standards. After vortexing for 1 min, tubes were incubated in water bath at 80 °C for 5 min with frequent shaking. After cooling tubes on ice, 1 ml of 3 N H_2_SO_4_ and 0.5 ml of 4-dimethyl-amino-benzaldehyde were added in succession into glass tubes followed by incubation in water bath at 70 °C for 16 min with slight shaking. The optical density of supernatant was measured at 560 nm. Hyp content per gram of tissue was determined according to the standard curve. Total collagen content was calculated by multiplication of the Hyp content by 7.5^22, 23^.

### Quantitative Real Time Polymerase Chain Reaction (qRT-PCR)

One piece of the left footpad skin sample including stratum corneum, epidermis, dermis, and a very thin layer of subcutaneous tissue, was collected and stored in RNAlater^®^ (Life Technologies, Grand Island, NY) at 4 °C for 24 h and then at −20 °C until total RNA isolation. Total RNA was isolated from footpad skin samples using MagMAX™-96 for Microarrays Kit (Life Technologies) after homogenization in Trizol^®^ (Life Technologies). One µg of total RNA, 11-mer oligo mix from Fluoresentric, and M-MLV Reverse Transcriptase (Life Technologies) were used to synthesize cDNA according to the manufacturer’s instructions. Levels of mRNA were measured by qRT-PCR using Applied Biosystems^®^ SYBR^®^ Green PCR Master Mix (Life Technologies) and a 7500 Fast Real-Time PCR System. Results were expressed as the level relative to the corresponding housekeeping gene *actin*. All primers (Table 2) were verified for efficiency (100% ± 10%) and linearity (r^2^ ≥ 0.99) of amplification.

**Table 2:** List of primers used for qRT-PCR.

Gene	Forward primer	Reverse primer
IL-1β	CAGCCCGTGGGCATCA	CTTAGCTTGTAGGTGGCGATGTT
TIMP3	CTCCAACTTCGGCCACTCA	CTTCCACCCTCTGGATGCA
TIMP4	TCATCTGCGATTCTGCTTTAGTG	GGCAGGAACCACCTTTTCAC
MMP13	TCTGACAGTCCCTATTCCTCTTGA	TCTGCAAACCGGAGGTCTTC
VEGF	AGAAAGGCCGGTACAAACCA	AGTGCTTTCTCCTCTCTGAGCAA
CD40	CCTGGTGATGCTGTGAATTG	CTTCTGTGTCGTTGCATTCAG
TNC	GCTCTCAAATTTCTCCTCCAGTCT	CCTTTTCAAAGCTGATGGAGTCTT
COL1A1	GCAGAATACTACCGGGCTGATC	TTTTCAGAGTGGCATCAACTTCA
COL3A1	ATGTGAAGGCTGGCTCAGTTG	GTCCCGGAAAGCCACTAATG
ITGA2	CCCGAATCTGGAACAGTACCTT	TGCCTCAGCAAAGAGTTGCA
ITGA3	GAGCGGCTCGACATCGA	AGCCACATGTCCTCAATGATGTA
Actin	CAACACAGTGCTGTCTGGTGGTA	ATCGTACTCCTGCTTGCTGATCC
TNFα	TGTTCTATGACCGCCCAGTTC	GACGTGTCACGATCATCTGGTT

### Statistical Analyses

All data were tested for normality and subjected to analysis of variance as a completely randomized design using the proc mixed procedure of SAS 9.4^24^. Each pen was used as the experimental unit for the analysis. Growth performance including BW, cBWG, cFI and cPI used the average data per pen. Gene expression and total collagen protein used individual measurement from one representative bird per pen. Area under the curve (AUC) for footpad lesion scores and relative mRNA levels of genes associated with FPD wound healing was calculated using the trapezoid method. Statistical differences among means were determined by using Duncan’s multiple-range test.

## Results

### Growth Performance

Growth performance of the broilers at 14, 28 and 42 d-of-age is summarized in Table 3. Both LTM and HTM improved BW, cBWG, cFI and cPI on d14 (*P* < 0.002); however, only the chickens that received HTM had higher BW, cBWG (*P* < 0.01) and cFI (*P* < 0.04) than NTM chickens on d28, and better cFCR (P < 0.03) than NTM on d42 (Table 3).

**Table 3:** Growth performance at 14, 28 and 42 d of age of broilers fed three supplemental trace mineral levels.

Item	BW (g)	cBWG (g)	cFI (g)	cFCR	cPI
Day 14					
NTM	0.474^b^	0.429^b^	0.509^b^	1.191	277.4^b^
LTM	0.499^a^	0.454^a^	0.534^a^	1.174	295.5^a^
HTM	0.504^a^	0.460^a^	0.545^a^	1.186	299.1^a^
SEM	0.0107	0.0105	0.0067	0.018	14.5
*P*-value	0.0002	0.0002	0.0023	0.3197	0.0226
Day 28					
NTM	1.768^b^	1.724^b^	2.308^b^	1.339	457.8
LTM	1.814^ab^	1.770^ab^	2.325^b^	1.313	484.8
HTM	1.859^a^	1.814^a^	2.399^a^	1.323	469.2
SEM	0.0309	0.0308	0.0268	0.0094	13.1
*P*-value	0.0189	0.0191	0.028	0.0517	0.3196
Day 42					
NTM	3.423	3.378	5.209	1.543^a^	509.2
LTM	3.482	3.437	5.221	1.518^ab^	527.2
HTM	3.577	3.533	5.312	1.505^b^	529.0
SEM	0.0997	0.0995	0.1119	0.0151	18.9
*P*-value	0.1495	0.1488	0.6273	0.0221	0.3252

^a,b^Means within a column with different superscripts differ at *P* < 0.05.

Reported standard error of the mean (SEM) is the average SEM of the individual least-square means.

### Footpad Lesions

**Figure 1:**
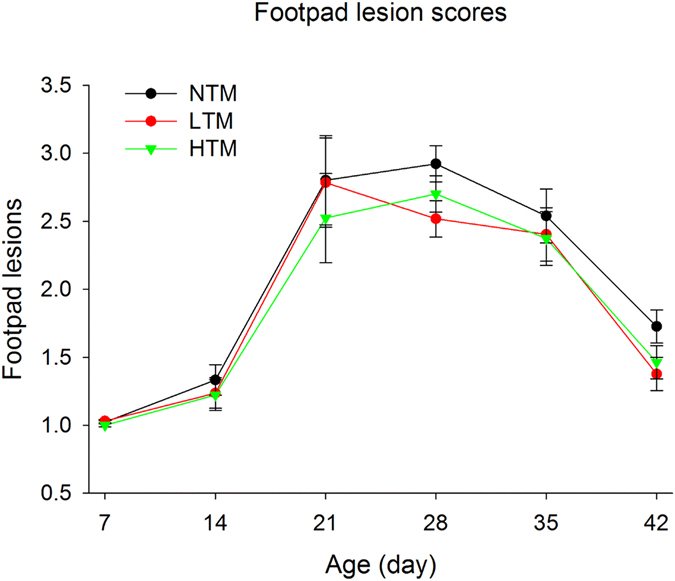
Footpad lesion scores of broilers fed three levels of TM at different ages. There was no interaction between treatments and age.

Figure 1 shows the results of footpad lesion scores among NTM, LTM and HTM treatments in broiler chickens at different ages. On d 21, 71% birds in all groups developed mild to moderate FPD, fresh and dry litter was top-dressed on existing litter on d21, 28 and 35 to promote FPD healing. TM did not alter litter moisture content in this study (Supplemental Table 1). Compared to NTM, LTM reduced (*P* = 0.06) FPD scores on d28, both LTM and HTM reduced (*P* = 0.08) FPD scores on d42 (Fig. 1). HTM reduced AUC of FPD lesion scores during d7–21 (*P* = 0.036), both LTM and HTM reduced AUC of FPD lesions scores during d21–42 (*P* = 0.043) and d7–42 (*P* = 0.074) (Table 4).

**Table 4:** Area under the curve (AUC) of footpad lesion scores in broilers fed three supplemental trace mineral levels during d 7 to 21, d 21 to 42, and d 7 to 42 of age.

Item	Treatment			SEM	*P*-value
	NTM	LTM	HTM		
AUC d7–21	21.99^a^	21.43^a^	15.14^b^	1.97	0.036
AUC d21–42	55.15^a^	43.22^b^	42.98^b^	4.37	0.043
AUC d7–42	76.31	64.70	66.45	4.70	0.074

^a,b^Means within a row with different superscripts differ at *P* < 0.05.

Reported standard error of the mean (SEM) is the average SEM of the individual least-square means.

### Wound healing markers

**Figure 2:**
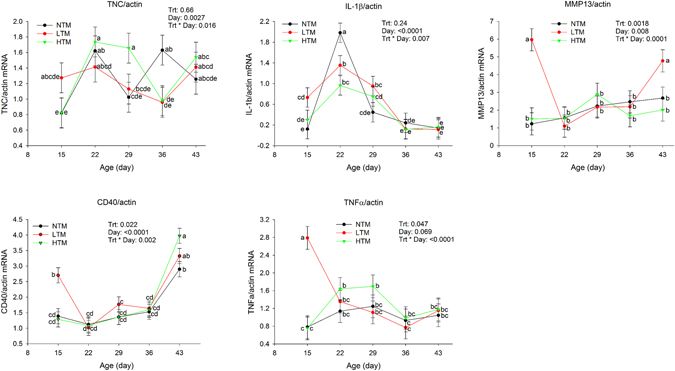
Gene expression of TNC, IL-1β, MMP13, CD40 and TNFα in footpad of broilers fed three levels of TM at different ages. There was interaction between treatments and age. Means with different letters differ at *P* < 0.05.

**Figure 3:**
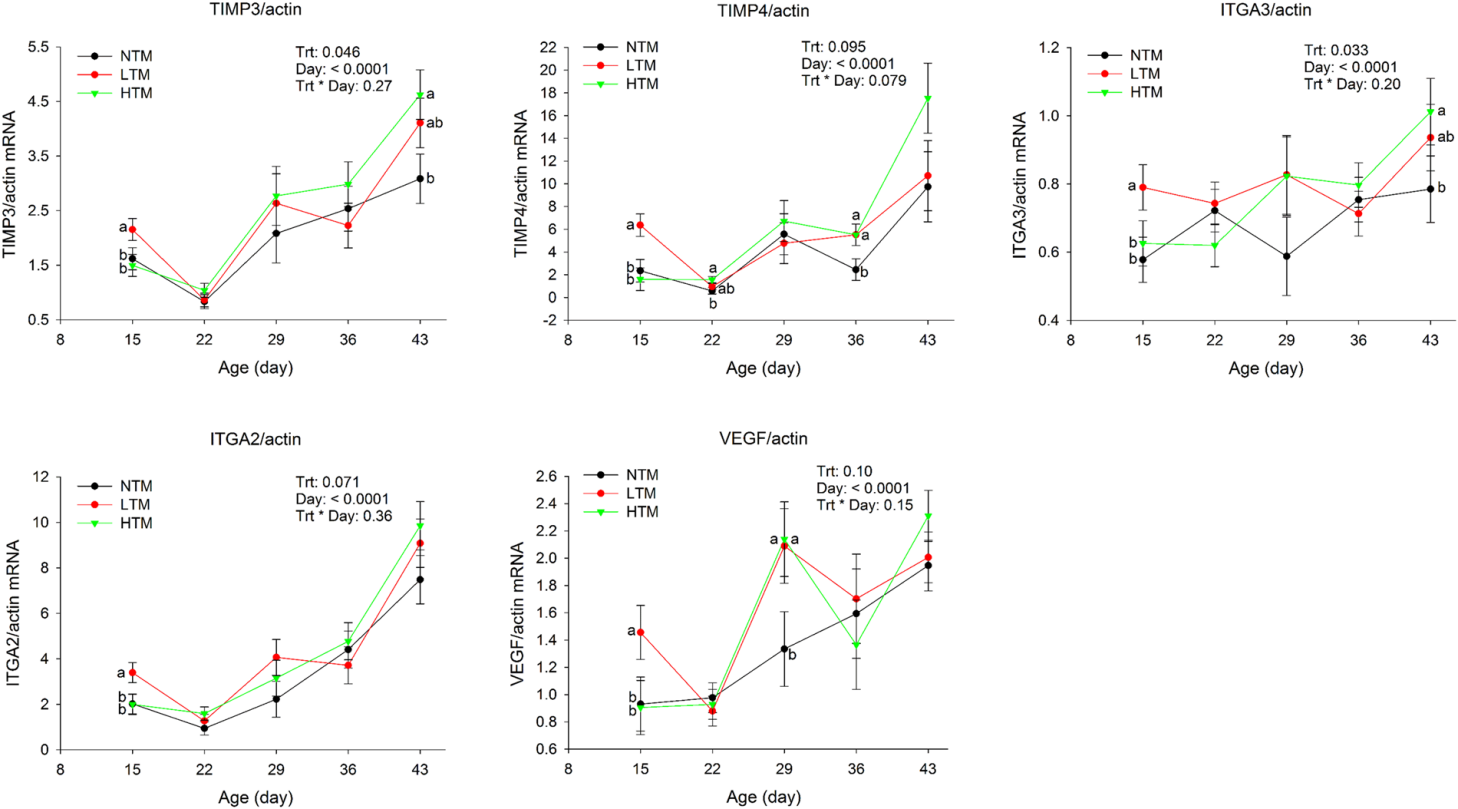
Gene expression of TIMP3, TIMP4, ITGA3, ITGA2 and VEGF fed three levels of TM. There was no interaction between treatments and age, treatment differences in birds at same age were shown in the graphs. Means with different letters differ at *P* < 0.05.

LTM increased (P < 0.05) the mRNA levels of matrix metallopeptidase 13 (MMP13) on d15 and d43, cluster of differentiation 40 (CD40), interleukin 1 beta (IL-1β), tumor necrosis factor alpha (TNFα), tissue inhibitor of metalloproteinase 3 (TIMP3), tissue inhibitor of metalloproteinase 4 (TIMP4), integrin alpha 2 (ITGA2), integrin alpha 3 (ITGA3) and vascular endothelial growth factor (VEGF) on d15 (Figs 2 and 3), and AUC of CD40 and MMP13 during d15–43 (Table 5).

**Table 5:** Relative mRNA levels of the genes potentially associated with footpad dermatitis wound healing as measured by Area under the curve in broilers fed three supplemental trace mineral levels during d15 to 43 of age.

Item	Treatment			SEM	*P*-value
	NTM	LTM	HTM		
TIMP3	44.65^b^	59.44^a^	65.13^a^	5.32	0.015
TIMP4	84.57^b^	140.93^a^	159.08^a^	27.19	0.036
MMP13	47.29^b^	88.98^a^	50.73^b^	8.56	0.001
ITGA2	90.01	97.59	95.47	11.09	0.854
ITGA3	18.59	21.49	20.47	0.92	0.111
VEGF	35.64^b^	45.07^a^	45.89^a^	3.98	0.010
CD40	40.32^b^	54.14^a^	46.85^b^	2.89	0.014
IL-1β	20.88	17.44	14.38	2.96	0.326
COL1A1	51.64	62.48	65.46	6.57	0.321
COL3A1	86.88	107.58	117.48	10.81	0.158
Collagen	189.68	218.65	190.94	10.91	0.119
TNC	42.16	29.96	31.69	7.28	0.226

^a,b^Means within a row with different superscripts differ at *P* < 0.05.

Reported standard error of the mean (SEM) is the average SEM of the individual least-square means.

HTM increased (P < 0.05) the mRNA levels of tenascin C (TNC) on d29, TIMP4 on d22, CD40, TIMP3 and ITGA3 on d43 (Figs 2 and 3).

Both LTM and HTM increased (P < 0.05) mRNA levels of VEGF on d29, TIMP4 on d36 (Fig. 3), AUC of TIMP3, TIMP4 and VEGF during d15–43 (Table 5), and reduced (P < 0.05) IL-1β on d22.

No differences were detected in the AUC of total collagen protein, type I alpha 1 collagen (COL1A1) and type III alpha 1 collagen (COL3A1) mRNA during d15–43 (Table 5) and their levels at individual time points (data not shown).

## Discussion

FPD is characterized by inflammation and necrotic lesions, ranging from superficial to deep, on the plantar surface of footpads and toes of chickens. Deep ulcers may lead to abscesses and thickening of underlying tissues and structures of the skin^1, 4^. Following a cutaneous lesion, the innate host defense initiates inflammation. This multifaceted process includes the cooperative efforts of different cell lineages during phases of proliferation, migration, matrix synthesis, and contraction, as well as the growth factors and matrix signals present at a wound site^25^. If the wound is not infected, the inflammatory response decreases and is substituted by angiogenesis and tissue remodeling^26^. Some of these cells, including immune and endothelial cells, keratinocytes, and fibroblasts, undergo changes in gene expression and phenotype^27^. In mammals, genome-wide transcriptional analysis tools have been developed to study the progression of changes during wound healing^13, 28^.

In the present study, high levels of TM improved growth performance on d14, d28 and d42 and reduced AUC of FPD lesion score during d7–21 and d21–43, low levels of TM improved growth performance on d14 and reduced AUC of FPD lesion score during d7–21. These results suggest that high levels of TM were slightly more effective than low levels of TM in improving growth performance and reducing footpad lesions.

To understand the role of TM in wound healing, the relative mRNA levels of 13 genes previously described to be involved in the development and wound healing of footpad lesions in chickens^19^ were measured at various time points in this study.

### MMP13 and TIMP

Lower levels of TM increased mRNA levels of MMP13 on d15 and d43. Lower and/or higher TM also increased mRNA levels of TIMP3 and TIMP4 on several days of the study, and AUC of TIMP3 and TIMP4 mRNA levels during d13–43. MMP13 is collagenase 3 that degrades collagen. TIMPs regulate extracellular matrix remodeling, especially cell migration in wound healing by modulating the activity of specific MMP^29^. TIMPs, especially TIMP3, also play an integral role in the regulation of inflammation^30^. In normal connective tissue, MMP and TIMP are balanced in order to maintain structural integrity. The balance between MMP and TIMP is also critical for the normal wound-healing process^31, 32^. The increase of TIMP3, TIMP4 and MMP13 indicates that active matrix remodeling was occurring in footpads, suggesting that TM improved cell migration and matrix remodeling as described previously^33–36^.

### Collagen and integrins

Collagen is the main and most abundant structural protein in the connective tissue of the extracellular space. It is also the primary protein component of structural tissues such as tendons, ligaments and skin in animals. Collagen contributes up to 35% of the whole-body protein content^13, 28, 37^. Type I collagen is the most abundant collagen in skin. Integrins are heterodimeric glycoproteins that contain non-covalently associated α and β subunits. They are expressed in basal layer keratinocytes *in vivo* and responsible for intercellular or cell–substrate adhesion and for sensing mechanical stress^38–40^. Integrin α2 (encoded by ITGA2) and integrin α3 (encoded by ITGA3) bind type I, II, III, and XI collagen^41^, forming a heterodimer with integrin β1 subunit, hence acting as a functional cellular receptor for type I collagen fibrils to facilitate both cell spreading on type I collagen matrix and contraction of type I collagen gel^35, 40^. In the present study, low and/or high levels of TM did not affect the amount of total collagen content, but increased the mRNA levels of ITGA2 and ITGA3. This finding further underlined the important role of TM in promoting collagen organization and remodeling by upregulating integrins during the re-epithelialization and cell migration phase of wound healing in chicken footpads.

### CD40

CD40, expressed by fibroblasts, regulates the communication between tissue fibroblasts and T cells, which is necessary for wound healing. Increase of CD40 gene expression by high TM on d43 and increase of CD40 gene expression on d15 and AUC of CD40 during d15–43 by low TM, indicate that TM may have improved wound healing by regulating the communication between fibroblasts and T cells.

### TNC and VEGF

TNC is produced by migrating and proliferating epidermis and plays an important role in wound healing. TNC gene expression is regulated by mechanical stress^42^, and it is expressed at high levels in wounds^44^. The mRNA levels of TNC were up-regulated by high levels of TM on d29 and down-regulated by both low and high levels of TM on d36, and were not different on d43. Tenascins are extracellular matrix glycoproteins that are abundant in the extracellular matrix of various organs and around healing of wounds^43, 44^. Induction of TNC gene expression on d29 suggests that TM promoted cell migration and proliferation during the early phase of wound repair.

In the present study, lower levels of TM increased VEGF expression on d15, and both low and high levels of TM increased VEGF expression on d29. VEGF is one of the most potent proangiogenic growth factors in the skin that functions in angiogenesis, formation of granulation tissue, wound closure and re-epithelialization^35^. Angiogenesis is required following the formation of granulation tissue to provide vascular support for the newly formed tissue during wound repair^45, 46^. Therefore, the amount of VEGF present in a wound can impact healing process. TM improved wound healing by increasing the levels of VEGF during the early wound healing phase. The reduction or lack of difference of TNC and VEGF gene expression after d29 suggests the decreased need of active wound repair, which indicates that footpad lesions are successfully healing, and is consistent with the reduction of footpad lesion scores.

### TNFα and IL-1β

Inflammation is required to remove injured tissue and cell debris during wound healing^47^, and this inflammatory phase typically lasts for the first 4 days post injury^48^. Proinflammatory cytokines, such as IL-1β and TNFα, are raised right after skin wounding^13, 28^. During normal wound healing, the highest levels of TNFα arise anywhere from 12 to 24 h after wounding^49^. After completion of the proliferative phase of wound healing, TNFα returns to basal levels^27^.

The mRNA levels of TNFα were increased in LTM treatment at d15 when the birds started to develop footpad lesions, but returned to basal levels by d22 and remained at basal levels afterwards. The mRNA levels of IL-1β were also increased in LTM treatment at d15, but were lower than control in both LTM and HTM treatments on d22, and were not different from control after d22. These results suggest that low levels of TM improved wound healing by promoting inflammation to remove injured tissue/cells during the inflammation phase (early wound healing phase, d15) and reducing further inflammation and tissue damage during the proliferation and maturation phase (late wound healing phase, d22 onwards), which helps to promote the wound healing process.

### Summary

There were no differences between low and high levels of TM in most of parameters measured in this study except for 1) growth performance where high levels of TM were slightly more effective on d28 and d43; 2) AUC of footpad lesion scores where high levels of TM were slightly more effective during d7–21 and d15–43; 3) gene expression of MMP13 and CD40 where low levels of TM were slightly more effective.

In summary, supplementation of TM not only improved growth performance, but also reduced footpad lesions by improving the wound healing process via promotion of collagen synthesis, deposition and organization, cell migration, matrix remodeling, angiogenesis; and regulation of inflammation. The role of individual minerals in FPD wound healing will be the subject of future studies.

## Electronic supplementary material

Supplemental table and figure
