# Supplementary material for: Impact of Trace Minerals on Wound Healing of Footpad Dermatitis in Broilers
Source: Sci Rep. 2017 May 15;7:1894. doi: 10.1038/s41598-017-02026-2 (PMC5432487; doi:10.1038/s41598-017-02026-2)

Impact of Trace Minerals on Wound Healing of Footpad Dermatitis in Broilers

Juxing Chen[1,*], Guillermo Tellez[2], Jeffery Escobar[1,#] & Mercedes Vazquez-Anon[1]

[1] Novus International, Inc. 20 Research Park Drive, St. Charles, MO 63304, USA. [2] Department of Poultry Science, University of Arkansas, Fayetteville AR 72701, USA.

[*]Correspondence should be addressed to
Dr. Juxing Chen
Novus International, Inc.
20 Research Park Drive,
St. Charles, MO, 63304, USA
Email: juxing.chen@novusint.com
Tel: 636-926-7418; Fax: 636-926-7449

[#]Current address: Elanco Animal Health, 2500 Innovation Way, Greenfield, IN, 46140, USA.

Supplemental Table 1. Litter moisture in the pens of broiler chickens at 6, 16, 20, 26 and 33 day of age.

Treatment	Day				
	**6**	**16**	**20**	**26**	**33**
NTM	17.0	38.2	46.2	38.2	42.6
LTM	19.1	38.9	45.4	38.9	41.3
HTM	20.2	34.2	45.0	34.2	37.1
SEM	2.3	3.6	1.8	3.6	2.5
P value	0.58	0.62	0.89	0.62	0.31

Supplemental Figure 1. Footpad lesions were examined for ulcer and scabs and scored using 5-points scoring system. Score 1 = no lesions, dermal ridges intact within central plantar footpad surface, with or without discoloration; Score 2 = mild lesions, dermal ridges not intact with small scab ≤ 2 mm in diameter on the central plantar footpad surface; Score 3 = moderate lesions with scab of 2-7 mm in diameter on the central plantar footpad surface; Score 4 = severe lesions with large scab ≥7 mm in diameter in the central plantar footpad surface but no scabs on the toes; Score 5 = most severe lesions with large scab ≥7 mm in diameter in central plantar footpad surface and small scabs on toes.

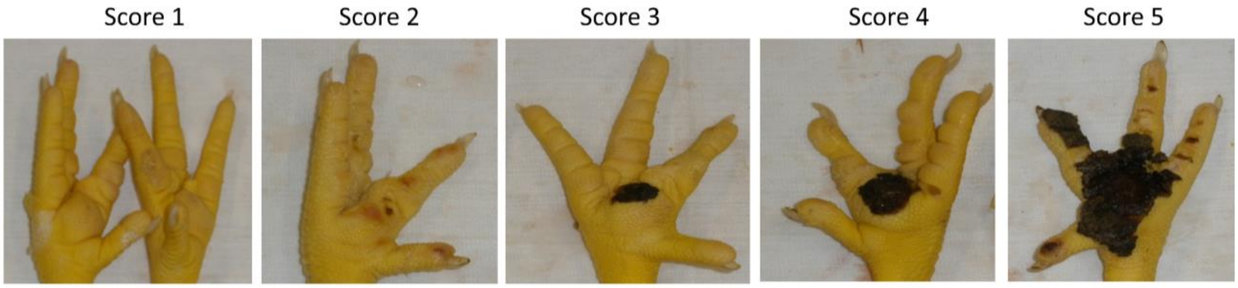

Supplement: Supplementary file 1 — Supplemental table and figure [file 41598_2017_2026_MOESM1_ESM.pdf]
